# Use of Encapsulation Technology to Improve the Efficiency of an Iron Oral Supplement to Prevent Anemia in Suckling Pigs

**DOI:** 10.3390/ani9010001

**Published:** 2018-12-20

**Authors:** Osmaly Churio, Emerson Durán, Sergio A. Guzmán-Pino, Carolina Valenzuela

**Affiliations:** Faculty of Veterinary Sciences, University of Chile, Santiago 8820808, Chile; osmaly@veterinaria.uchile.cl (O.C.); emerson.duran@ug.uchile.cl (E.D.); sguzmanp@uchile.cl (S.A.G.-P)

**Keywords:** anemia, encapsulation, erythrocytes, ferrous sulphate, piglets

## Abstract

**Simple Summary:**

In animal nutrition, encapsulation technologies have been poorly explored, even though they may improve oral iron supplementation in suckling pigs. We hypothesized that the encapsulation of an oral supplement could enhance iron bioavailability for suckling pigs. We compared the levels of biomarkers for iron nutritional status among three groups of animals: piglets receiving oral encapsulated supplements, individuals receiving oral non-encapsulated supplements (as control group), and animals who were supplemented by parenteral route (strategy currently used in pig production systems). All treatments prevented anemia at weaning; however, only oral treatments prevented the first stage of anemia, which is iron depletion. In conclusion, piglets supplemented with encapsulated iron showed a higher concentration of serum ferritin and, therefore, had better iron reserves.

**Abstract:**

The objective of this study was to develop an encapsulated iron supplement for oral ingestion and to determine its effect on the iron nutrition status of suckling pigs. Encapsulated and non-encapsulated iron supplement was prepared. Seventy-two neonatal piglets were assigned to three experimental groups: (1) parenteral group (gold standard treatment), which received one dose of parenteral iron (200 mg), 2 days of age, (2) “non-encapsulated” group (as a control group), which received 4 oral doses of unencapsulated iron supplement at 2, 7, 12 and 17 days of age, and (3) “encapsulated” group, which received 4 oral doses of encapsulated iron supplement on the same days. The encapsulated and unencapsulated iron supplements contained 65.2 and 65.0 mg/iron/dose, respectively. Parenteral treatment was not sufficient to ensure an adequate iron nutritional state in piglets at the end of the lactation period, showing iron depletion (serum ferritin: 8.4 µg/L). In contrast, oral supplementation prevented the development of iron depletion. Higher serum ferritin values were observed in the encapsulated (19.9 µg/L) compared to the unencapsulated group (17.4 µg/L) (*p* = 0.020). In conclusion, the use of four oral doses of an encapsulated iron supplement prevents iron deficiency anemia and its previous stages in suckling pigs.

## 1. Introduction

Iron deficiency anemia is the main form of malnutrition in suckling piglets raised under intensive conditions, with a multicausal origin. Failure to supplement iron over their first days of life results in poor production performance [1,2], altered cognition [3], increased susceptibility to illness [4], and even death [5]. For decades, the most used method for delivering iron supplements during early life stages has been through intramuscular injections of 100 or 200 mg of dextran iron [1,6]. However, recent observations have led to the realization that these injections were not sufficiently effective for preventing iron deficiency. Researchers have reported finding piglets whose iron reserves were depleted [7] or deficient after weaning, and many were clearly anemic [2]. Other authors have also observed that the iron homoeostasis of piglets is altered by using such elevated doses of dextran iron, leading to cases of toxicity [6], as well as the activation of hepcidin secretion, that is, the secretion of a peptide which acts as a negative regulator of iron absorption and reuse [8]. Additionally, this form of iron supplementation has been linked to muscle tissue damage and locomotor problems [9].

The oral route is another option for the administration of iron supplements. However, many attempts at using this route have failed to achieve satisfactory results in suckling piglets. Several reasons explaining these results have been proposed. For example, there is a poor expression of non-heme iron receptors and transporters in suckling piglets during the first week of life [6]. In addition, a low bioavailability of commonly used non-heme iron sources (ferrous sulfate, ferrous fumarate, among others) has been described [10,11]. On the other hand, organic sources of iron, such as heme iron, have a greater bioavailability [11]. Unfortunately, there are few studies that use heme iron as an oral supplement in pigs [7,11], and, for this reason, we used heme iron from bovine erythrocytes in this work. In addition, it was recently reported that the use of bovine hemoglobin as a dietary source of heme iron for iron-deficient piglets improved the iron nutrition status of the animals [12]. Other explanations may include the need for an administration at elevated and repeated doses due to the low bioavailability of non-heme iron [13] and, finally, the unsatisfactory organoleptic properties of iron, which result in animals rejecting common supplements [14].

Encapsulation technology could solve some of the aforementioned problems. For example, a greater iron bioavailability and lower rejection rates have been reported for fortified foods and supplements fed to humans and rodents [15,16]. Some information on the use of encapsulation technology for domestic animal nutrition has already been published [17]. However, there are few reports about the use of this technology to improve the bioavailability of iron in pigs. The use of this technology for the improvement of oral iron supplementation in suckling piglets has been reported in a previous study conducted by our group [7]. However, this study did not address certain pending questions on whether encapsulation could effectively enhance the bioavailability of iron in piglets.

In a previous study conducted in our group [7], maltodextrin was used as the encapsulating material, mainly due to its low cost, which is important for its application in animal production. It was also used because maltodextrin is a material that is compatible with spray drying technology, which is an encapsulation technique with industrial applications. Finally, good results were obtained in the previous study with this encapsulating material, which justify the reutilization of maltodextrin in the design of the present work. In this study, it was hypothesized that encapsulation technology could enhance iron’s bioavailability. This could be manifested by better levels of biomarkers indicating the nutritional status of iron in animals receiving oral encapsulated supplements than in those that are given non-encapsulated oral supplements. Therefore, the aim was to develop an oral encapsulated iron supplement and to determine its effect on the iron nutrition status of suckling pigs.

## 2. Materials and Methods

### 2.1. Material

We selected food-grade maltodextrin with 20 DE (dextrose equivalent), purchased from Prinal S.A. (Santiago, Chile), for the encapsulating material. The nucleus of the microparticles comprised ferrous sulphate heptahydrate (FS) (Merck S.A., Darmstadt, Germany) as a non–heme iron source and atomized bovine erythrocytes (BE) (Licán Alimentos S.A., Santiago, Chile) as a heme iron source. 

The iron content of each iron source was determined by atomic absorption spectrophotometry using a GBC 905AA atomic absorption spectrophotometer (GBC Scientific Equipment, Braeside, Australia) following the directives for acid digestion method 999.11 [18].

### 2.2. Iron Encapsulation

The iron was encapsulated in maltodextrin microparticles by spray drying. We prepared maltodextrin solution in deionized water at 40% *w*/*v*. Then, each nucleus material was dispersed separately in the maltodextrin solution at a concentration of 20% *w*/*v* by using a magnetic agitator. Homogenized blends were then immediately fed to a B–290 mini spray dryer (BÜCHI Labortechnik AG, Flawil, Switzerland). With respect to the operational parameters, we chose to set the inlet and outlet air temperatures at 140 ± 5 °C and 95 ± 5 °C, respectively. The air flow, rate of feeding, and atomization pressure were 500 L/h, 8 mL/min, and 20 psi, respectively. The resulting microparticles for every blend were collected in plastic containers and stored at room temperature. 

The microparticles were observed by scanning electron microscopy. The microparticles were mounted on a cylindrical aluminum stub using double-sided tape to sputter-coat them with gold twice, at 20 kV in an argon atmosphere using a PELCO 91000 sputter coater unit (Ted Pella, Inc., Redding, CA, USA). We then examined our samples under a LEO 1420 VP scanning electron microscope (LEO Electron Microscopy, Cambridge, UK) at an accelerating voltage of 25 kV. 

We determined the total iron content for microparticles using a GBC 905AA atomic absorption spectrophotometer (GBC Scientific Equipment, Braeside, Australia) following the directives for acid digestion method 999.11 [18]. Spectrophotometric measurements were then assessed against a standard curve plotted at a wavelength (λ) of 248.3 nm for a 1000 µg/mL commercial iron standard solution (J.T. Baker, Phillipsburg, NJ, USA).

### 2.3. Supplement Compounding

To develop the encapsulated supplement, we mixed both types of microparticles in a 1:1 ratio, that is, 1 g of FS microparticles with 1 g of BE microparticles (contributing 65.2 ± 2.5 mg of iron), and we added 3 mL of distilled water. This suspension was then loaded into 10 mL syringes.

We also prepared a control supplement that contributed a similar amount of iron (65.0 ± 1.8 mg) and was based on the same iron sources, but these were not encapsulated. For the elaboration of the non-encapsulated supplement, each iron source (heme and non-heme) was dissolved in deionized water without maltodextrin and then this dispersion was atomized in the spray dryer, using the same parameters. 

### 2.4. In vivo Study

#### 2.4.1. Animals

All experimental procedures were approved by the Bioethics Committee of the Faculty of Veterinary and Animal Sciences, University of Chile (certificate N 18132-VET-UCH). The experiment was performed at a commercial pig farm (Región Metropolitana, Chile). A population consisting of seventy-two male and female 1-day-old piglets, weighing 1.5 to 1.8 kg, were selected and allocated to 6 sows (12 piglets per litter). The piglets consumed only milk during the study.

Piglets from each litter were randomly assigned to three experimental groups: (1) a parenteral group (*n* = 24): piglets injected with 200 mg of dextran iron (Duplafer®, Veterquímica, Chile) into their thigh muscles at 2 days of age; (2) unencapsulated group (as a control group) (*n* = 24): comprising piglets supplemented with 4 oral doses of unencapsulated iron supplement at 2, 7, 12, and 17 days of age; and (3) encapsulated group (*n* = 24): piglets supplemented with 4 oral doses of the encapsulated iron supplement at 2, 7, 12, and 17 days of age. The body weight of the animals was recorded at the beginning (day 1) and at the end (day 21) of the experimental period. The average daily gain was also calculated.

We maintained a parenteral group because it is considered the pig industry’s “gold standard” treatment [7,19]. It was not possible to maintain a negative control group, that is, without treatment, for ethical reasons.

**Figure 1:**
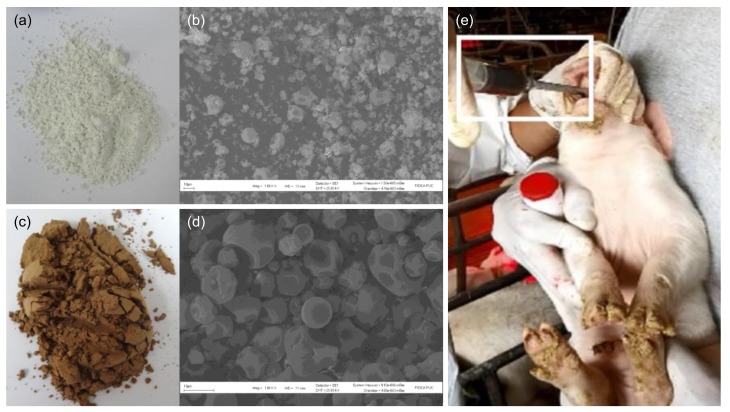
Scanning electron micrograph showing the appearance and morphology of ferrous sulphate (FS) microparticles (**a**) and (**b**) and bovine erythrocytes (BE) microparticles (**c**) and (**d**), respectively. Appearance of the encapsulated supplement and delivery method used with piglets (**e**).

To minimize product loss, piglets were individually grabbed and held by their belly while the supplement was directly poured within their mouth via a curved drench syringe (Figure 1E). Each piglet received a total dose of 260 mg of iron, which is sufficient to meet their requirements for the whole lactation period up to weaning at 21 days of age [20].

#### 2.4.2. Iron Status

To assess the iron status, blood samples of 2 mL were drawn from the piglets by jugular venepuncture at days 1 and 21. Red blood cells (RBC), hemoglobin (Hb), hematocrit (Ht), mean corpuscular volume (MCV) (Model ZBI; Coulter, Hialeah, FL and Cell-Dyn 1700; Abbott Diagnostic, Abbott Park, IL, USA), and serum ferritin (SF) (Pig ferritin, ELISA Kit; Cusabio, Hubei, China) were determined. Cut-off values for biomarkers were those used by Antileo et al. [7]: Red blood cell (<5.3 10^6^ × mm^3^), hematocrit (<32%), mean corpuscular volume (<50 fL), hemoglobin (<9 g/dL), and serum ferritin (<12 µg/L).

#### 2.4.3. Behavioral Observations

The reactions of the piglets were observed for five minutes immediately after administering the supplement. Signs of rejection behaviors were recorded, such as sideways head movement, repeated exposure of tongues, mouth rubbing against wood shavings or other surfaces, self-isolation, or delays in returning to suckle [14].

### 2.5. Statistical Analysis

The iron content of microparticles was analyzed by Student’s t-test using Statistix 8 software (Analytical Software 2003, Tallahassee, FL, USA), with a level of significance of 0.05. The body weight and the average daily gain of the animals were analyzed with ANOVA by using the GLM procedure of SAS (version 9.0; SAS Institute Inc., Cary, NC, USA). The iron status biomarkers were analyzed between days of treatment and for the different treatments with ANOVA by using the GLM procedure of SAS (version 9.0; SAS Institute Inc., Cary, NC, USA). Mean values are presented as least square means adjusted by Tukey. The α level used for the determination of significance was 0.05. The following mathematical model was used:

Yij = µ + αi + βj + εij
(1)

where Y is the iron status biomarker, µ is the general mean of all observations, α is the effect of treatment (parenteral, unencapsulated, and encapsulated supplement), β is the effect of the age of the piglets at the moment of the test (1 and 21 days) and ε is the random error.

## 3. Results

### 3.1. Supplement

The different iron sources were used to prepare microparticles. The FS presented an iron content of 142 mg/g and bovine erythrocytes (BE) of 2.5 mg/g. Iron content values were greater for FS microparticles (66.5 ± 0.7 mg/g) than for BE microparticles (0.77 ± 0.11 mg/g) (*p* < 0.001), as expected, due to the different iron concentration of iron sources.

The microparticles made from ferrous sulphate (FS) were characterized as greenish (Figure 1A), whereas BE microparticles were brown (Figure 1C). All microparticles were spherical (Figure 1B,D), although FS microparticles had a more rugged surface (Figure 1B). Scanning electron microscopy photographs at the same magnification levels showed that FS microparticles were smaller (Figure 1B,D). 

Both microparticles were soluble in distilled water, resulting in a homogeneous dispersion suitable for the administration as a liquid oral supplement (Figure 1E, see white box).

### 3.2. Effects of Encapsulated Oral Iron Supplements on the Iron Status of Piglets

No differences (*p* > 0.05) were observed in the body weight of the animals at the beginning of the study between the groups (Table 1). Table 2 shows the change in the indicators of iron status over the course of the supplementation study. At the beginning of the study, all the piglets presented an optimal iron status. At the end of the study, no animals of either group demonstrated anemia. However, the piglets of the parenteral group presented serum ferritin levels below the cut-off point. Therefore, a one-dose parenteral treatment is not sufficient to ensure an adequate iron nutritional state for weaning piglets (21 days old). Even though piglets are not anemic following such routine treatment, they do present iron depletion, which has been described as the first stage of anemia. This condition did not influence the productive parameters of the pigs, as no significant differences in body weight or average daily gain were observed between the groups at the end of the study (Table 1).

Both oral supplements efficiently maintained an optimum state of iron by weaning time; all biomarkers assessed remained above the cut-off value (Table 2). No significant differences were found in red blood cell, hematocrit, mean corpuscular volume, and hemoglobin biomarker levels between the encapsulated and the unencapsulated treatments. However, serum ferritin was higher for the group provided with the encapsulated supplement at the end of the study (Table 2) (*p* < 0.001). Both groups provided with oral supplementation presented higher values of iron biomarkers compared to the parenterally supplemented group, with the exception of red blood cell count (Table 2). 

### 3.3. Behavioral Observations

Finally, we observed a decrease of behavioral manifestations with the administration of encapsulated supplement. Serious discomfort was attributed to piglets assigned to the unencapsulated iron group. Especially common behaviors were sharp, sideways head movements, tongue protrusion, mouth rubbing on wood shavings, and delaying suckling for 2–4 min longer compared to those piglets who received an encapsulated supplement. 

## 4. Discussion

### 4.1. Iron Supplements

The analysis of both types of microparticles (BE and FS) has revealed that the iron content greatly differed between the two types. This difference is explained by the abundant organic matrix present in whole erythrocytes, which are used to prepare BE microparticles [21], in contrast with the inorganic origin of the iron source used in FS microparticles [22]. Although BE microparticles contribute only small amounts of iron to the supplement formula, they fulfil an important role in pig diets because of the greater bioavailability of heme iron [11,19], which is also absorbed using a different mechanism from non-heme iron [12].

### 4.2. Effects of Parenteral and Oral Iron Supplements on the Iron Status of Piglets

In line with recent reports from other authors [2,7], the results of this study on in vivo supplementation showed that the parenteral supplement was not sufficiently effective to ensure that piglets sustained their iron levels at an optimum nutritional state by the time of weaning. Specifically, monitoring the serum ferritin concentration showed that the iron deposits of these piglets were depleted at that point. Many studies examining the role of supplements in combating anemia in pigs do not report serum ferritin levels; therefore, assessments based on hematologic results alone have indicated an optimum nutritional status for iron. In reality, iron deficiency stages are likely being underestimated for suckling and weaning piglets. Determining serum ferritin was regarded as the most objective method to determine the nutritional iron status by Smith et al. [23], in contrast to a diagnosis based on the concentration of hemoglobin or other biomarkers, which may present retarded alterations due to the half-life of erythrocytes.

As it was expected, no changes were observed in the productive parameters of animals at the end of the study, since changes in body weight have only been observed in deficiency and anemia states [2]. We demonstrated that oral treatments resulted in better nutritional states of iron in piglets. These results might be explained by the enhanced organic response to lower doses, even though they were administered more frequently. This finding agrees with reports from Starzyński et al. [8], who administered two smaller doses of dextran iron (70 mg) to suckling piglets and compared the outcomes to a single, higher dose of iron at farrowing time. They found that the latter increased plasma hepcidin-25 levels quite significantly. Conversely, the lower doses favored iron homoeostasis by drastically reducing hepcidin levels in plasma.

Other studies have investigated alternative oral supplementation schemes in suckling piglets and have found an anti-anemic effect. However, these approaches required using higher doses than those used in this study. For example, Svoboda and Drábek [24] used a total iron dose of 400 mg, which doubles established nutritional requirements for suckling piglets [20]. In another study by Loh et al. [13], piglets received 32 mg of iron lactate per mL of drinking water, starting on their third day of life and up to their weaning time (28 days). Those piglets were also offered food containing 1 g/kg of ferrous sulfate from the seventh day onwards.

In this work, the observed anti-anemic effect was achieved using four doses of low iron concentration. The results can be explained by several reasons. The first is that the mixture of heme and non-heme iron sources in the supplement are absorbed using two different mechanisms, the heme protein-1 carrier receptor and the enterocyte’s divalent metal transporter [12,17]. The second is that the heme iron in piglets provides greater bioavailability than non-heme sources [11]. Finally, the third reason is that heme iron potentiates intestinal absorption of non-heme iron in piglets [25].

### 4.3. Effect of Encapsulation of Iron Sources on Iron Status of Piglets

A positive aspect found in this study was that the encapsulation process improves serum ferritin values in weaned pigs. The fact that the enhanced effect of iron encapsulation was only reflected in serum ferritin values and not in other biomarkers could be due to the high specificity of this protein for iron deficiency anemia and its ability to rapidly predict iron absorption because it can distinguish differences in iron storage [23]. Serum ferritin has a high correlation with iron absorption, which makes it highly sensitive to dietary iron changes [26,27]. Conversely, other measures of iron status, such as hemoglobin or other biomarkers related to erythrocytes, reflect changes in iron nutrition status but more slowly [23]. 

However, the effect of the encapsulation on the iron nutrition status of the pigs was not as expected. A possible explanation for the low effect on the iron bioavailability observed for encapsulated iron is that maltodextrin could be an unsuitable material for enhancing iron bioavailability. This outcome could be a consequence of maltodextrin’s elevated solubility in aqueous solutions [22,28] and simulated solutions of the gastrointestinal content [29]. Microparticles could be soluble throughout, thus resulting in the continuous release of their iron content. As a consequence, iron might have been equally subject to precipitation, negative interactions with other nutrients or diet ingredients, or redox reactions that resulted in its limited absorption. Another reason could be that the chemical structure of maltodextrin comprises multiple glucose molecules joined together by α-glucosidic bonds [30]. These bonds are readily degraded by enzymes that are found in the small intestine. Looking forward to future studies, it is important to be aware that the selection of an encapsulating material is a critical point during the design stage of the experiment. A major requirement is that materials should retain as much iron as possible under gastric conditions but should then release most iron under intestinal conditions, ideally in a controlled manner.

The application of four doses of iron during lactation has a greater economic value and requires more handling of animals. However, this management may reduce iron depletion at weaning, which could also reduce economic losses due to lower performance. For example, it was established that an anemic pig weighs 0.82 kg less than a non-anemic pig [2]. In fact, the productive and economic repercussions of iron depletion are currently unknown.

### 4.4. Effect of Encapsulation on the Behavior of Pigs When Consuming Oral Supplements

Encapsulation led to a lower incidence of rejection behaviors when animals were fed the supplement. In contrast, the group that received the unencapsulated supplement displayed rejection behaviors, likely associated with the unpleasant taste of both iron sources. Human studies [31] have reported that participants described the taste as metallic or blood-like for FS and BE, respectively. In piglets, the feeling of discomfort that animals experienced may explain their delay in returning to nurse and it may also explain the reduction of resting time that has been described in other studies on oral iron supplementation [14].

## 5. Conclusions

We successfully encapsulated high concentrations of both heme and non-heme iron sources within maltodextrin microparticles. The use of these microparticles as an oral supplement could represent another strategy to reduce anemia and its previous stages in suckling pigs. Nonetheless, it is recommended that other encapsulating wall materials might be evaluated with the goal of enhancing the uptake of iron within the intestines of young animals.

## Figures and Tables

**Table 1:** Mean and standard error of mean (SEM) of body weight and average daily gain of piglets subjected to different iron supplementation protocols at the beginning (day 1) and at the end of the experiment (day 21).

Productive Parameters	P	US	ES	SEM	*p*-Value
Live weight (day 1)	1.609	1.618	1.622	0.017	0.8525
Live weight (day 21)	6.270	6.271	6.269	0.187	0.9999
Live weight gain	0.222	0.221	0.221	0.009	0.9992

Treatment: P: parenteral, US: Unencapsulated supplement, ES: Encapsulated supplement.

**Table 2:** Biomarkers for the iron nutrition status expressed as mean and standard error of mean (SEM) for piglets subjected to different iron supplementation protocols.

Biomarkers ^†^	Parenteral			
	Day 1	Day 21	SEM	*p*-Value
Red blood cell (10^6^ × mm^3^)	6.1	5.9	0.08	0.0855
Hematocrit (%)	32.2 ^a^	30.0 ^b1^	0.42	0.0007
Mean corpuscular volume (fL)	52.5 ^a^	50.1 ^b1^	0.59	0.0084
Hemoglobin (g/dL)	10.8 ^a^	9.3 ^b1^	0.16	<0.001
Serum ferritin (µg/L)	21.4 ^a^	8.4 ^b1^	0.72	<0.001
	**Unencapsulated supplement**			
Red blood cell (10^6^ × mm^3^)	6.2	6.1	0.08	0.3334
Hematocrit (%)	31.5	32.3 ^2^	0.55	0.2873
Mean corpuscular volume (fL)	52.1	53.2 ^2^	0.65	0.2318
Hemoglobin (g/dL)	10.9	11.3 ^2^	0.23	0.1958
Serum ferritin (µg/L)	18.6	17.4	0.79	0.3028
	**Encapsulated supplement**			
Red blood cell (10^6^ × mm^3^)	6.1	6.1	0.08	0.5660
Hematocrit (%)	32.4	32.8 ^2^	0.30	0.3052
Mean corpuscular volume (fL)	52.9	54.2 ^2^	0.69	0.2230
Hemoglobin (g/dL)	11.2	11.6 ^2^	0.23	0.2558
Serum ferritin (µg/L)	20.8	19.9 ^3^	0.76	0.4076

^†^ Cut-off values of biomarkers: red blood cell (<5.3), hematocrit (<32), mean corpuscular volume (<50), hemoglobin (<9), serum ferritin (<12) [7]. ^a,b^ Means followed by different letters in superscript in a column indicate differences between days within each treatment (*p* < 0.05). ^1,2,3^ Means followed by different numbers in superscript in a column indicate differences at 21 days within each treatment (*p* < 0.05).
